# Ozone Exposure of a Weed Community Produces Adaptive Changes in Seed Populations of *Spergula* *arvensis*

**DOI:** 10.1371/journal.pone.0075820

**Published:** 2013-09-26

**Authors:** Jennifer B. Landesmann, Pedro E. Gundel, M. Alejandra Martínez-Ghersa, Claudio M. Ghersa

**Affiliations:** 1 Laboratorio Ecotono, INIBIOMA-CONICET, Universidad Nacional del Comahue, Bariloche, Río Negro, Argentina; 2 Cátedra de Ecología, Facultad de Agronomía (UBA), IFEVA-CONICET, Ciudad Autónoma de Buenos Aires, Buenos Aires, Argentina; Beijing Forestry University, China

## Abstract

Tropospheric ozone is one of the major drivers of global change. This stress factor alters plant growth and development. Ozone could act as a selection pressure on species communities composition, but also on population genetic background, thus affecting life history traits. Our objective was to evaluate the consequences of prolonged ozone exposure of a weed community on phenotypic traits of *Spergula* *arvensis* linked to persistence. Specifically, we predicted that the selection pressure exerted by high ozone concentrations as well as the concomitant changes in the weed community would drive population adaptive changes which will be reflected on seed germination, dormancy and longevity. In order to test seed viability and dormancy level, we conducted germination experiments for which we used seeds produced by *S. arvensis* plants grown within a weed community exposed to three ozone treatments during four years (0, 90 and 120 ppb). We also performed a soil seed bank experiment to test seed longevity with seeds coming from both the four-year ozone exposure experiment and from a short-term treatment conducted at ambient and added ozone concentrations. We found that prolonged ozone exposure produced changes in seed germination, dormancy and longevity, resulting in three *S. arvensis* populations. Seeds from the 90 ppb ozone selection treatment had the highest level of germination when stored at 75% RH and 25 °C and then scarified. These seeds showed the lowest dormancy level when being subjected to 5 ºC/5% RH and 25 ºC/75% followed by 5% RH storage conditions. Furthermore, ozone exposure increased seed persistence in the soil through a maternal effect. Given that tropospheric ozone is an important pollutant in rural areas, changes in seed traits due to ozone exposure could increase weed persistence in fields, thus affecting weed-crop interactions, which could ultimately reduce crop production.

## Introduction

Global change is affecting the pattern of species distribution, communities composition and ecosystems functioning [1]. The study of strategies that allow species to persist is essential for our understanding of global change impacts [2,3]. Much of the research conducted has focused on the effects of global warming on species, in order to predict their capacity to cope with increasing temperatures, CO_2_, drought and pollutants [2,4,5]. Among them, tropospheric ozone is becoming an important global change stress factor to be considered [6-8]. Even though the incidence of this atmospheric pollutant and greenhouse gas is increasing [9,10], there is scarce knowledge about its effects on plant life history traits involved in population persistence and growth.

Tropospheric ozone is a stress factor for biological systems whose adverse effects on plants have been broadly documented. This is particularly true in rural areas where its concentration may be high as a consequence of the elevated amount of hydrocarbons emitted by vegetation [7,10-12]. Attention has been mainly focused on the direct effects of ozone at species level, such as changes in biomass production, visible injury symptoms as well as the underlying molecular mechanisms [7,12-15]. Recently, however, more interest has been focused on the study of effects at community level, focusing on species richness and diversity [8,16]. The existence of phenotypic variability in species sensitivity to ozone suggests a potential capacity of ozone for driving species evolutionary changes [17-20]. Nevertheless, there are still no long-term experimental studies addressing this particular question.

Persistence in new environments can be achieved by rapid evolution towards resistant phenotypes, high phenotypic plasticity, phenological and demographic changes, high dispersal rates, or complex combinations of all these processes [2,21-23]. Under the present scenario of global change, those species that do not have any of the previously listed abilities or traits could be at risk of extinction [2,24]. Seed characteristics and behaviour have received special consideration since they are essential life history traits determining population dynamics and persistence, particularly in annual species [5,25,26]. Seed traits such as germination rate, dormancy and longevity, are involved in the dynamics of soil seed banks and seedling establishment [5,22,25,27]. For instance, since dormancy and germination are directly influenced by temperature, species persistence in soil seed banks may be threatened by increasing temperatures [25]. Similarly, tropospheric ozone might drive changes in seeds through a selection pressure on the population or through a maternal effect during seed development, both affecting seed quality and behaviour. Hence, the documented reduction in seed quality of oilseed rape and germination in seeds of paper birch, both produced under conditions of increased ozone, constitute evidence of maternal effects [28,29]. However, no studies have yet assessed the consequences of long-term ozone exposure on seed traits, and particularly, differentiating selection from maternal effects.

Variations in seeds germination, dormancy and longevity driven by increased tropospheric ozone could cause changes in species composition and relative abundance through differential adaptive microevolution, altering species interactions and ecosystem properties. Particularly in rural areas, this could affect weed-crop interaction and crop production, especially if weed species become more persistent in the field [7,30]. *Spergula* *arvensis* L. (corn spurry) is a summer annual herb that depends on seed production and fitness-related traits (e.g. dormancy and rapid germination) to persist in vegetation communities. It is distributed worldwide, particularly in cereals, although it can also be found in other crops and grasslands ( [31,32] and cites therein). Its seeds usually germinate in spring after overwintering [32] and they present high levels of primary dormancy physically imposed by seed coats, allowing them to remain alive in the soil bank for a very long time [33]. Large and persistent soil seed banks have been reported in cultivated or abandoned fields [34,35]. The objective of this study was to elucidate whether prolonged exposure of a weed community to episodic and high ozone concentrations could result in adaptive changes in *S. arvensis* populations. We focused on seed traits that can be crucial for species fitness in novel ecological scenarios. We hypothesized that the selection pressure exerted by increased ozone concentration and the concomitant changes on the overall weed community, drive adaptive evolution in *S. arvensis*. More precisely, we predict differences among populations in functional seed traits related to soil seed bank persistence. In order to test this hypothesis, we evaluated performance of *S. arvensis* populations selected under three contrasting ozone treatments during 4 years [8], under laboratory and field experiments.

## Materials and Methods

### Ozone selection treatments

**Figure 1:**
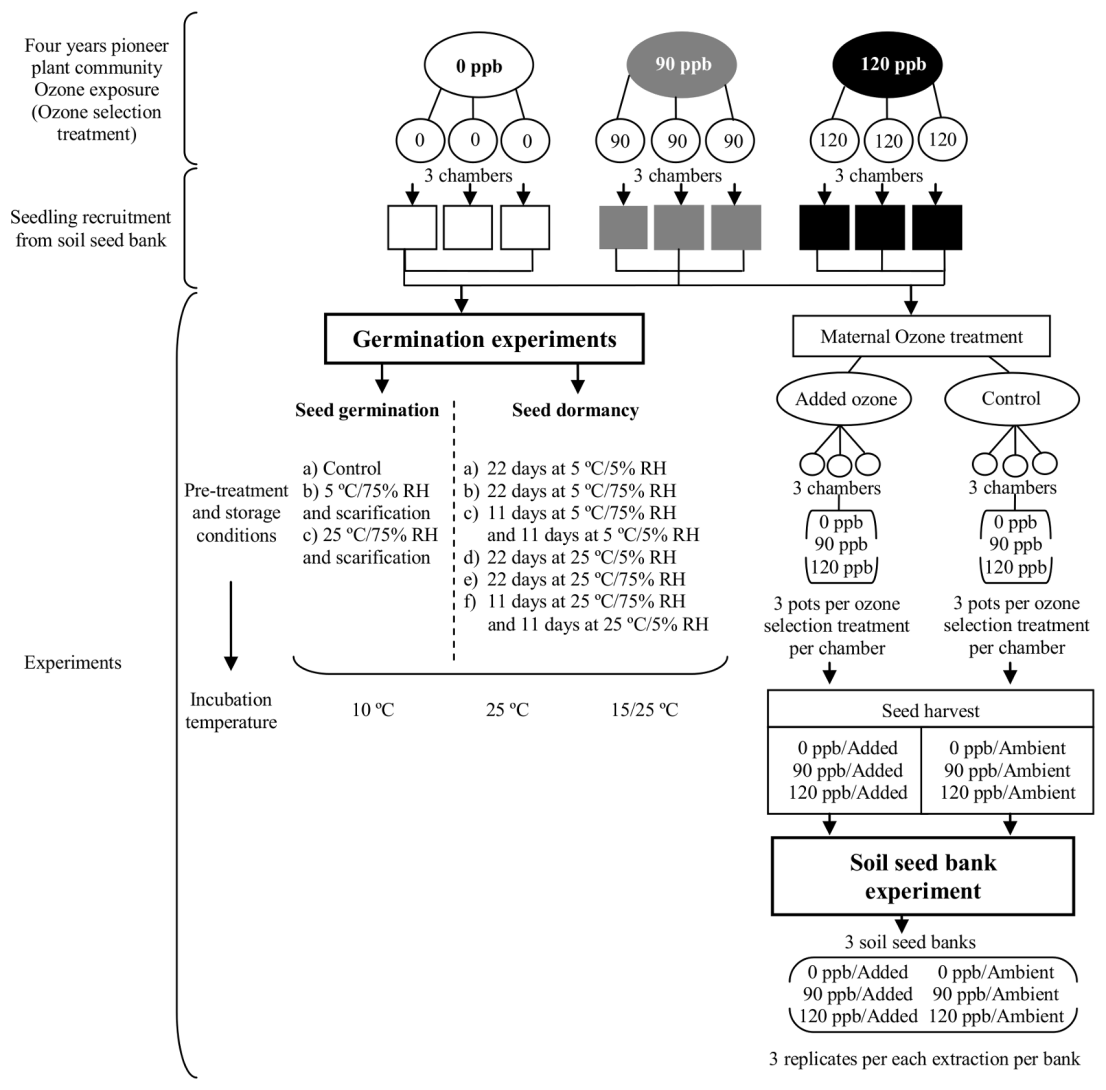
Overview of the history of ozone exposure and the experimental designs. Starting from a four-year exposure of a weed community under three ozone selection treatments (0, 90 and 120 ppb) [8]; the subsequent sowing of the soil seed bank samples, coming from each ozone selection treatment replication, in nine plots; the *Germination* *experiments* (Seed germination and Seed dormancy experiments) carried out with *S. arvensis* seeds harvested from the plots; and the *Soil* *seed* *bank* *experiment* developed with seeds harvested from plants exposed to ambient and added ozone (control and added ozone treatment respectively; maternal ozone treatments).

The populations of *S. arvensis* used in this work were selected in a long-term ozone exposure experiment carried out in Corvallis, Oregon, at the US Environmental Protection Agency Laboratory, Western Ecology Division (for details see [8]). A weed community was exposed to ozone for four consecutive years (Figure 1). Three ozone concentration regimes: 0 ppb (control), 90 ppb and 120 ppb (hereafter referred to as ozone selection treatments) were randomly assigned to nine open-top chambers. The ozone profile consisted of an episodic pattern, characterized by variations in the daily peak concentration, along 28-day cycles. All plants were allowed to reproduce and disperse seeds naturally in the plots within each chamber. After the experiment, soil containing seeds of the resulting weed communities from each ozone selection treatment replicate, was stored separately at ambient temperature in sealed plastic boxes. After two years of storage, samples of these soil seed banks were brought to the Agronomy Faculty, University of Buenos Aires (Argentina) to carry out the experiments in this work. No specific permissions were required for fieldwork in this study because it was carried out on lands owned by the Agronomy Faculty, University of Buenos Aires (Argentina). There are lands in this Faculty specifically destined for research studies fieldwork. The field studies did not involve endangered or protected species.

Nine field plots were placed in an experimental field at the University of Buenos Aires (34°35´ S, 58°35´ W) filled with sterilized organic soil. 420 cm^3^ soil seed banks samples from each ozone selection treatment replication were randomly sown in one of the nine plots (Figure 1). Pollen and seed flow among plots and from outside the experiment were prevented by surrounding each plot with a plastic wall. Plots were periodically irrigated. *S. arvensis* was one of the most abundant species present in all plots. It produced a large number of seeds which were harvested, threshed and stored for two years at 10 °C before being used in the *Seed germination* and *Seed dormancy experiments*.

### Seed germination experiment

Given that *S. arvensis* seeds present dormancy imposed by hard coats [36], we firstly applied a scarification treatment to determine the seeds general viability condition. Seeds were subjected to the following pre-treatments during 22 days: storage at 75% RH and 5 °C followed by scarification (i), and storage at 75% RH and 25 °C followed by scarification (ii) (Figure 1). Three samples with 50 seeds per ozone selection treatment replication were assigned to each pre-treatment. Each sample of seeds was placed in a 2 ml eppendorf, and all the eppendorfs assigned to one pre-treatment were placed within a plastic box. In each box, 75% RH was provided by a saturated NaCl solution (31.7 g NaCl in 88.1 ml H_2_O [37]) contained in four open top glass jars. In the free space inside the plastic box, the open eppendorfs were carefully fixed to a small rack. Both plastic boxes were placed in germination chambers at 5 °C and 25 °C respectively. After 22 days of storage, seeds were dried at ambient temperature for three days and then they were softly pressed with sandpaper, a scarification treatment for removing physical dormancy [36]. A control treatment was carried out with three samples of 50 seeds per ozone selection treatment stored under dry conditions at 10 °C.

Seed germination was then tested under three incubation temperatures: constant 10 or 25 °C, and alternating 15/25 °C (cycles of 12h). Nine samples (one per ozone selection treatment replication) were assigned to each incubation temperature provided by different growth chambers. Seeds from each eppendorf were sown on a filter paper moistened with 3 mL of distilled water in a Petri dish. Germination, as radicle protrusion, was recorded every 3 days during a period of 27 days. Germinated seeds were counted and removed from the dish. A sub-sample of non-germinating seeds, from each pre-treatment, was cut with a scalpel under a magnifying glass to check for viability. Those that were firm and healthy looking were considered viable and dormant, thus allowing separation of dead from alive seeds. The small number of seeds that did not germinate after the scarification pre-treatments were dead, particularly under the most stimulating incubation temperatures, whereas in the control, the majority of the non-germinated seeds were alive and dormant.

### Seed dormancy experiment

Patterns of seed dormancy release, as affected by the ozone selection treatments, were explored by modifying the after-ripening conditions and testing germination of the three *S. arvensis* seed populations under different incubation temperatures [32,38]. Seeds were exposed for 22 days to one of six storage conditions resulting from the combination of two different temperatures (5 and 25 °C) and three levels of relative humidity (22 days at 5% RH; 22 days at 75% RH; 11 days a 75% plus 11 days at 5% RH) (Figure 1). Three samples per ozone selection treatment replication, with 50 seeds each, were subjected to one of the six storage conditions. The 5% RH was provided by a saturated ZnCl solution (128 g in 61 ml H_2_O [37]). After 22 days of exposure, seed germination was tested under the same incubation temperatures as in the *Seed germination experiment* (Figure 1). Once again, seeds were cut at the end of the experiment, to test for viability of the non-germinated seeds. Most of the seeds which did not germinate under the least stimulating incubation temperature were alive and dormant. In turn, the few seeds which did not germinate under the most stimulating incubation temperatures were dead.

### Soil seed bank experiment

In order to differentiate evolutionary from maternal effects, *S. arvensis* plants from the three ozone selection treatments were grown under ambient or added ozone concentration and viability dynamics of the produced seeds was evaluated under soil bank condition (Figure 1). Plants were obtained by germinating seeds from each ozone selection treatment, collected in the field plots described above. Eighteen plants from each ozone selection treatment were transplanted individually into 250 cm^3^ pots and randomly distributed in six 8 m^3^ open-top chambers with crystal PVC walls mounted on a metal structure, at the Agronomy Faculty, University of Buenos Aires [39,40]. Plants were grown until reproductive stage, when they received the ozone fumigation treatments (hereafter referred to as maternal ozone treatments). Three chambers were used for control treatment (ozone ambient air) and three for added ozone treatment (charcoal filtered air with added ozone). Ambient air was pumped through an activated charcoal filter into all chambers for 3 hours around midday during 4 days. Ozone concentration in ambient air ranged between 0 and 20 ppb, the typical low value prevailing in the area due to titration of ozone with NO [41]. In added ozone treatment chambers fumigation increased ozone concentration to 40-70 ppb, a regime approximately 2,5-3 fold greater than that of the control treatment and similar to ozone concentrations recorded in many polluted regions of the world. Nevertheless, these exposures were not intended to simulate ambient conditions but rather to characterize the response of plants and seeds to air pollutant concentrations that produce obvious adverse growth effects. Ozone was produced by a spark discharge-type ozone generator and mixed with ambient air before it reached the chambers [39]. Ozone was monitored using a Model 450 Ozone Monitor API-Teledyne Instrument (Teledyne Advanced Pollution Instrumentation San Diego, CA). Average maximum and minimum temperatures during the experimental period were 27.9 and 16.8 °C, respectively. Differences between temperature inside and outside the chambers never exceeded 1.5 °C. At the end of the exposure period, the plants were placed in a greenhouse until seeds ripened, when they were carefully harvested. Three samples of 30 seeds per each ozone selection treatment (0, 90 and 120 ppb), coming from both maternal ozone treatments, were put into 3 x 7 cm nylon mesh bags and randomly placed in one of three 5 cm deep soil seed banks. Then, the bags were covered with soil and a plastic roof was placed over each bank to prevent rainfall from entering. Once a month, along seven months between summer and autumn, three samples per ozone selection treatment, of both maternal ozone treatments, were randomly extracted from the soil seed banks. Immediately afterwards, seeds samples were sown on a filter paper moistened with 3 mL distilled water placed in a Petri dish and incubated at 15/25 °C alternating temperature. After radicle protrusion, number of germinated seeds was recorded every two days until no further germination was observed. Non-germinated seeds were subjected to a Tetrazolium Test [42] to determine their viability. Thus, we obtained the total number of alive seeds per sample for every extraction. Seed water content and temperatures in each soil bank were assessed along the whole experimental period (See Appendix S1 and Figure S1 for methodology and data obtained).

### Data analysis

Analyses of data for the *Seed germination* and *Seed dormancy experiments* were performed by means of generalized linear models (glm package) assuming a binomial function of error structure and a logit link function with the R software [43,44]. The response variable was the proportion of germinated seeds per sample (number of germinated seeds in relation to total seeds). In the *Seed germination experiment*, ozone selection treatment (0, 90 and 120 ppb), pre-treatments (5ºC/75% RH + scarification; 25ºC/75% RH + scarification, and control) and incubation temperature (10, 25 and 15/25 °C) were used as independent variables, while in the *Seed dormancy experiment*, ozone selection treatment, storage temperature (5 and 25 °C), storage relative humidity (22 days at 5% RH, 22 days at 75% RH, and 11 days at 5% RH followed by 11 days at 75% RH), and incubation temperature (10, 25 and 15/25 °C) were used as independent variables. In both cases, the full models were first considered and then, they were simplified by removing non-significant terms until the minimum adequate models were obtained [43]. The statistic significance of factors was tested by deviance analysis. Due to interactions between storage conditions and incubation temperatures, separated analyses were performed within each storage condition to test for the effect of ozone selection treatment and incubation temperature.

Dynamics of seed viability in the *Soil seed bank experiment* was analyzed using mixed generalized linear models assuming a binomial response variable (live or dead seed) and time (days), ozone selection treatment (0, 90 and 120 ppb), maternal ozone treatment (control and added ozone) as independent variables. The bank was considered as a random effect. The analysis was performed using lme package of R software [43,44]. The same procedure of model selection was applied. Finally, separate analyses were performed to evaluate the effects of ozone selection treatment and time within the maternal ozone treatments.

## Results

### Seed germination experiment

**Figure 2:**
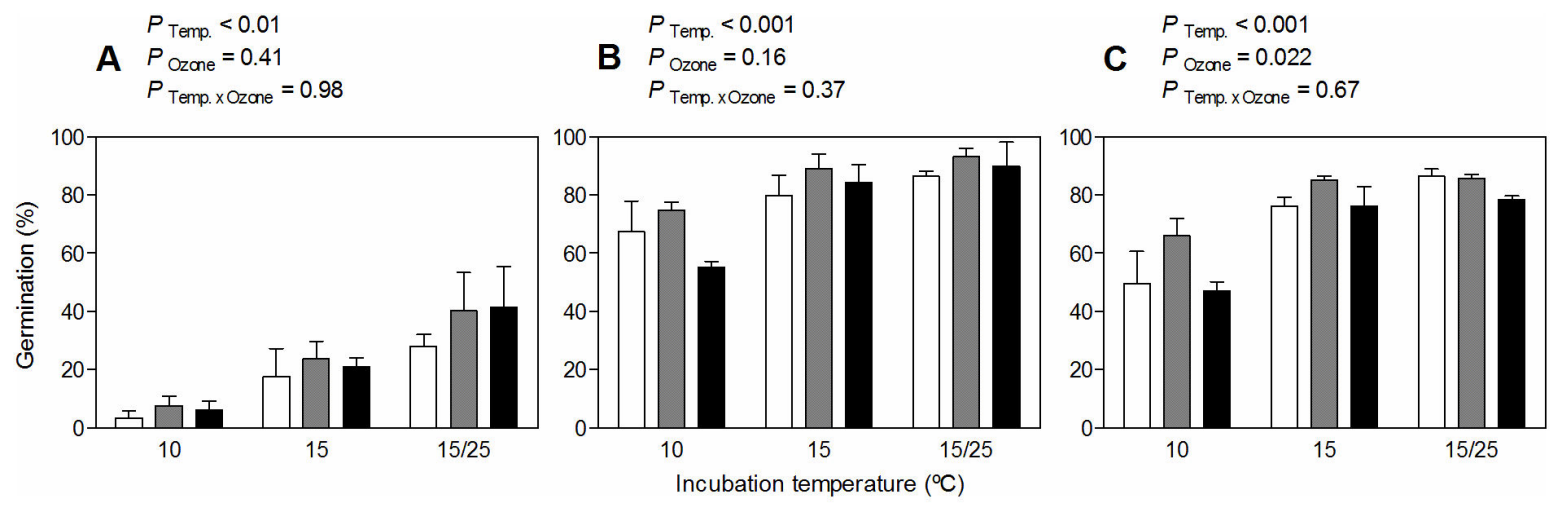
Germination (%) of *Spergula* *arvensis* seeds for each ozone selection treatment, pre-treatment and incubation temperature. White, grey and black bars represent germination of seeds (n = 3, ± SE) for 0, 90 and 120 ppb ozone selection treatment, respectively, after 22 days of exposure to pre-treatments: storage at 10 °C and dry condition (control) (A), storage at 5 °C and 75% RH followed by scarification (B), and storage at 25 °C and 75% followed by scarification (C); and then incubated at 10, 15 and 15/25 °C. Above each plot, *P-values* indicate the effects of Incubation Temperature (Temp.), Ozone selection treatment (Ozone) and the interaction (Temp. xOzone) within each Pre-treatment.

Seed germination response to pre-treatments differed among seed populations corresponding to the different ozone selection treatments (Table 1). The minimum adequate model for the overall *Seed germination experiment* considered the main effect of ozone selection treatment and the double interaction between pre-treatment and incubation temperature. Ozone selection treatment showed a marginal effect on seed germination (*P* = 0.062), whereas the effect of incubation temperature depended on the pre-treatment (*P* < 0.001). Control treatment showed the lowest proportion of germinated seeds compared with those pre-treatments that involved seed scarification (Figure 2). Nonetheless, the differential germination observed between 10°C (where germination was curtailed) and 15/25 °C (where germination was stimulated) was higher in the control than in the pre-treatments involving scarification (Figure 2). Almost full germination was obtained on scarified seeds incubated under 15/25 °C (Figure 2b and 2c).

**Table 1:** Analysis of Deviance (F test) for the minimum adequate model which describes the effects of Ozone selection treatment, Pre-treatments and Incubation temperature on *Spergula* *arvensis* seed germination.

**Source**	***Df***	***F***	***P***
Ozone selection treatment ¹	2	5.55	**0.062**
Pre-treatment ²	2	378.11	< 0.001
Incubation temperature ³	2	73.33	< 0.001
Pre-treatment x Incubation temperature	4	50.24	**< 0.001**

¹ 0, 90 and 120 ppb; ² storage at 10 °C and dry condition (control), storage at 5 °C and 75% RH followed by scarification (i) and storage at 25 °C and 75% RH followed by scarification (ii); ³ 10, 15 and 15/25 °C.

Analysis performed within each pre-treatment showed a significant effect of ozone selection treatment when seeds were stored at 75% RH and 25 °C and then scarified (Figure 2c). Specifically, germination of seeds from the 120 ppb ozone selection treatment was the lowest of the three populations, while germination of the 90 ppb ozone selection treatment was the highest (Figure 2c). Storage at 75% RH and 5 °C followed by scarification generated the highest amount of germinated seeds under the three incubation temperatures, but there were no differences between ozone selection treatments (Figure 2b). Finally, incubation temperature showed a consistent effect on seed germination (*P* < 0.001).

### Seed dormancy experiment

**Figure 3:**
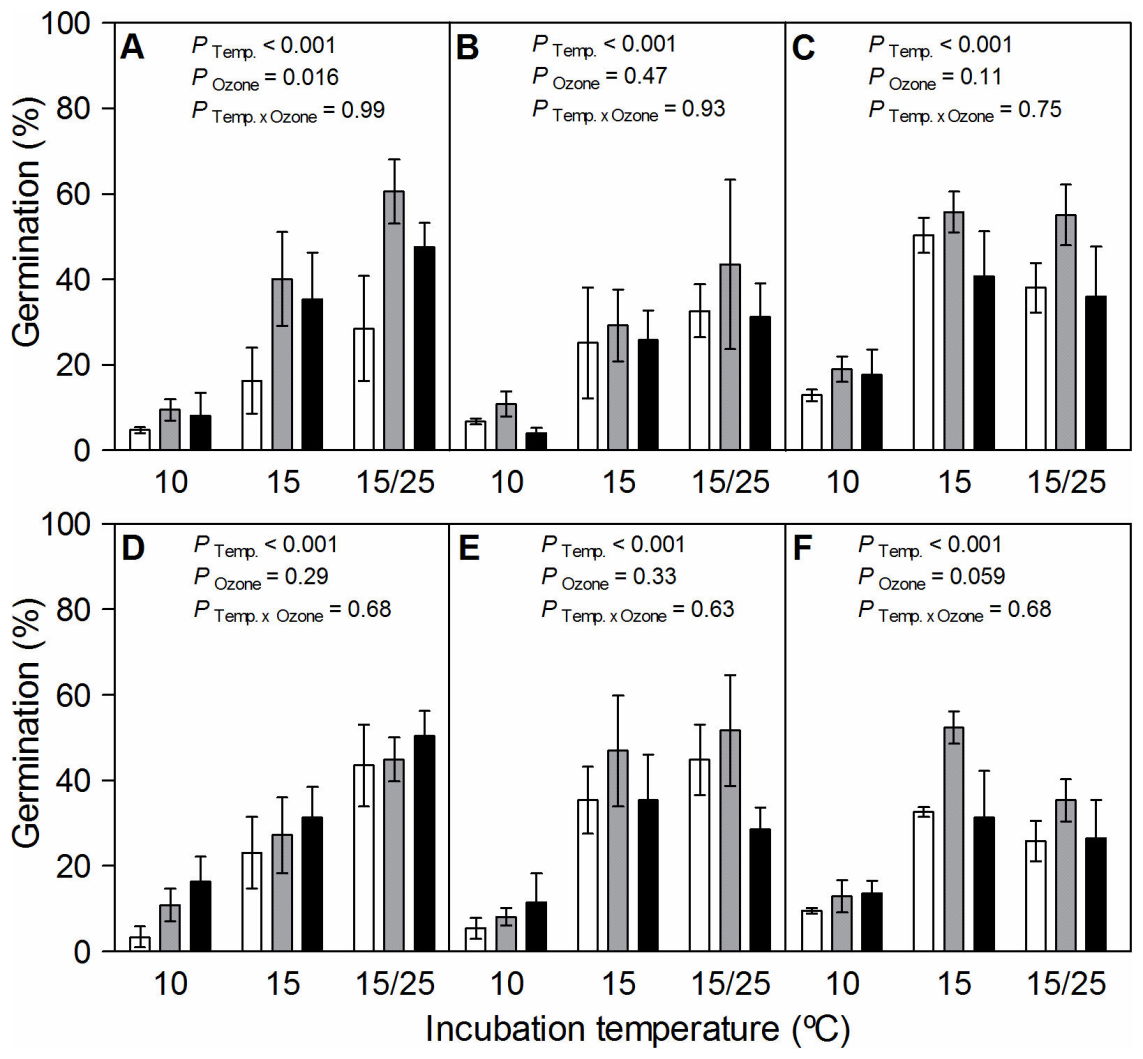
Germination (%) of *Spergula* *arvensis* seeds for each ozone selection treatment **, storage condition and incubation temperature**. White, grey and black bars represent germination of seeds (n = 3, ± SE) for 0, 90 and 120 ppb ozone selection treatment, respectively, after being exposed for 22 days to storage conditions combining two temperatures (panels A, B and C: 5 °C, and panels D, E and F: 25 °C) and three relative humidity regimes (panels A and D: 5% RH; panels B and E: 75% RH; panels C and F: 11 days at 75% and 11 days at 5% RH) and incubation temperatures (10, 15 and 15/25 °C). Above each plot, *P-values* indicate the effects of Incubation Temperature (Temp.), Ozone selection treatment (Ozone) and the interaction (Temp. xOzone) within each storage condition.

The general pattern of seed germination indicated variations in dormancy release among the seeds coming from the different ozone selection treatments when subjected to different storage conditions. The minimum suitable model for the *Seed dormancy experiment* included three double interactions: ozone selection treatment by relative humidity at storage, storage temperature by storage relative humidity, and storage relative humidity by incubation temperature (Table 2). Nonetheless, while the double interaction between ozone selection treatment and storage relative humidity was marginal (*P* = 0.086), the other two were significant (*P* < 0.05) (Table 2). Moreover, there was a strong effect of incubation temperature on seed germination (*P* < 0.001). The proportion of germinated seeds at 10°C was always lower than at 15°C. The difference between 15 °C constant and 15/25 °C alternating temperatures depended on the previous combination of temperature and relative humidity at storage (Figure 3).

**Table 2:** Analysis of Deviance (F test) for the minimum adequate model that describes the effects of Ozone selection treatment, Storage temperature, Storage relative humidity, Incubation temperature and interactions on *Spergula* *arvensis* seed germination.

**Source**	***Df***	***F***	***P***
Ozone selection treatment ¹	2	35.17	< 0.001
Storage temperature ²	1	0.49	0.614
Storage relative humidity ³	2	8.99	0.099
Incubation temperature ^4^	2	454.57	< 0.001
Ozone selection treatment x Storage relative humidity	4	15.94	**0.086**
Storage temperature x Storage relative humidity	2	21.95	**0.004**
Storage relative humidity x Incubation temperature	4	37.18	**0.001**

¹ 0, 90 and 120 ppb; ² 5 and 25 °C; ³ 5% RH, 75% RH, 75%/5% RH^4^; 10, 15 and 15/25 °C.

In general, seed germination differences between the ozone selection treatments depended on storage condition. In fact, analyses conducted within each storage condition, detected significant effects associated to ozone selection treatment in two of them: 5 °C and 5% RH (*P* = 0.016), and 25 °C and 75% followed by 5% RH (*P* = 0.059). Under these conditions, seeds from the 90 ppb ozone selection treatment presented the lowest overall dormancy level. This population had the highest proportion of seed germination under the three incubation temperatures (Figure 3a and 3f), being superior after storage at 5 °C and 5% RH, under 15/25 °C alternating temperature (Figure 3a). On the other hand, in spite of the fact that seeds from the 0 and 120 ppb ozone selection treatments presented a higher overall dormancy level, their germination response patterns were largely influenced by the incubation temperature. Seeds from the 120 ppb ozone selection treatment presented a higher level of germination after storage at 5 °C and 5% RH, while that of the 0 ppb ozone selection treatment was significantly lower after the same storage condition (Figure 3a).

### Soil seed bank experiment

**Figure 4:**
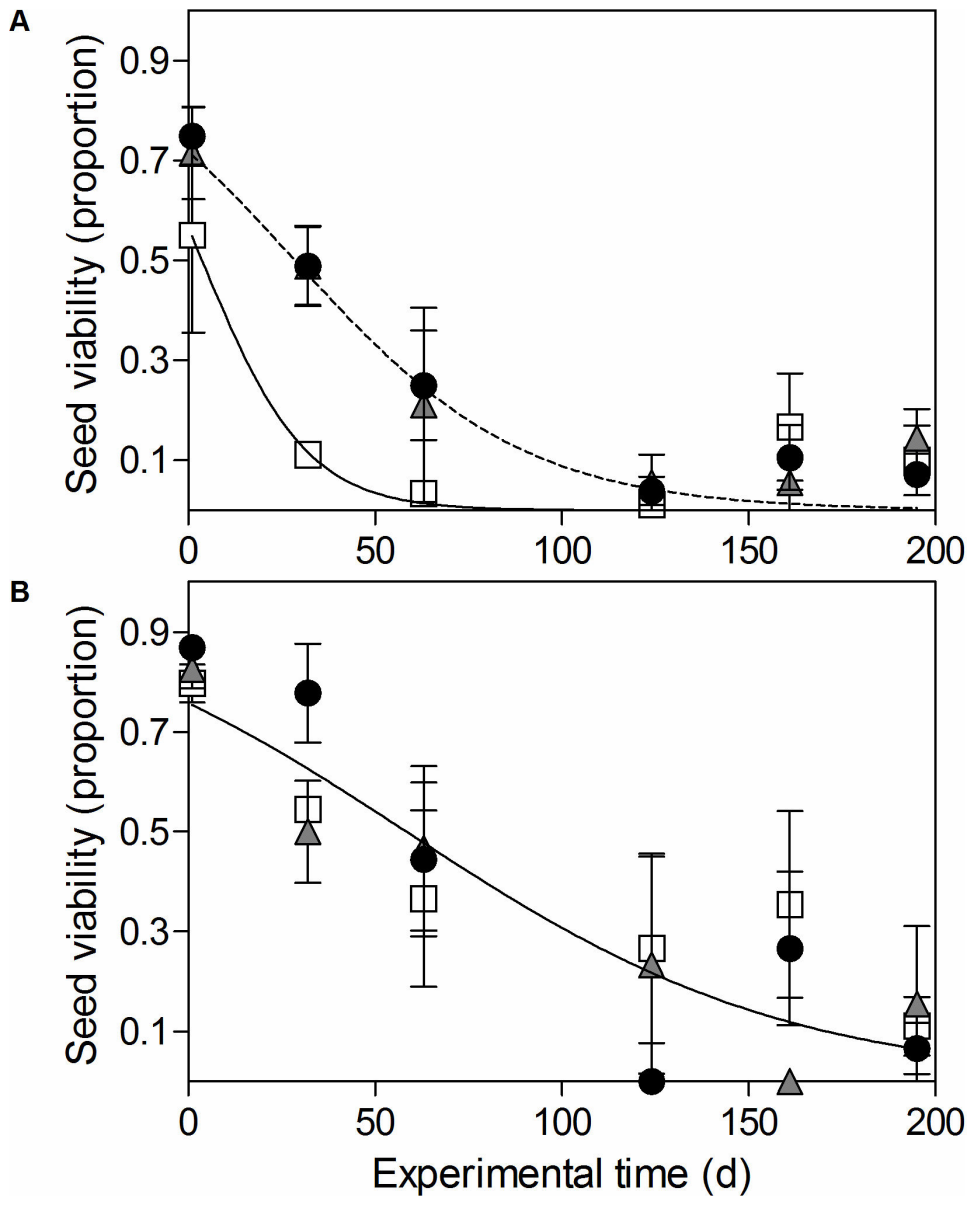
*Spergula* *arvensis* seeds viability for ozone selection and maternal ozone treatments related to burial time. White, grey and black symbols represent viability for seeds of the 0, 90 and 120 ppb ozone selection treatments, respectively, that were grown under ambient ozone concentration (A) or added ozone concentration (B), in relation to days of burial time. Values are means of three samples ± SE.

Seed viability dynamics in the soil bank was found to be dependent on both the ozone selection treatment and the mother plant exposure to ozone. The minimum suitable model that described seed viability in relation to experimental time in the soil bank included two double interactions: ozone selection treatment by maternal ozone treatment, and ozone selection treatment by time. However, the interacting effect between ozone selection treatment and maternal ozone treatment was not significant (*P* = 0.327), while ozone selection treatment effect depended marginally on the experimental time (*P* = 0.0624). Importantly, the maternal ozone treatment had a significant effect on seed viability dynamics (*P* < 0.001) (Table 3). When plants grew under ambient ozone, seeds from the different ozone selection treatments had a different viability dynamics in the soil (Figure 4a). And although the statistical differences were marginal, seeds from the control selection treatment (i.e. 0 ppb) showed a marked decrease in viability compared to those seeds from populations selected under higher ozone concentrations (90 and 120 ppb), while there was no difference between seeds produced under these two latter conditions (Figure 4a). On the other hand, seeds produced by mother plants exposed to an enriched ozone atmosphere (i.e. added ozone treatment) presented no difference between seeds coming from 0 ppb and either 90 ppb or 120 ppb, and there was a general increased seed viability irrespective of the ozone selection treatment (Figure 4b).

**Table 3:** Analysis of Deviance (F test) for the minimum adequate model for the effects of Time, Ozone selection treatment, Maternal ozone treatment and interactions on *Spergula* *arvensis* seed viability.

**Source**	***Df***	***F***	***P***
Time ¹	1	67.48	**<0.0001**
Ozone selection treatment²	2	1.10	0.3352
Maternal ozone treatment ³	1	12.49	**<0.0001**
Time x ozone selection treatment	2	2.87	0.0624
Ozone selection treatment x Maternal ozone treatment	2	1.13	0.3271

¹ Time was considered as a continuous variable with 6 dates; ² 0, 90 and 120 ppb; ³ ambient and added ozone concentration.

## Discussion

Selection pressure exerted by ozone and the concomitant changes on the overall weed community drove phenotypic changes in *S. arvensis* populations in germination, dormancy and longevity of seeds. Differences in these behavioural seed traits were found between ozone selection treatments, meaning that the long-term ozone selection pressure resulted in three differentiated populations of *S. arvensis* seeds. Furthermore, the exposure of plants to ozone increased seed persistence in the soil through maternal effect. This suggests that ozone appears to have two different effects: it exerts selection pressure on specific population traits and it modifies seed behaviour by means of a mother-plant mediated effect (i.e. acclimation). Abundant evidence proves that stress factors act as selection drivers of resistant genotypes [17,23] while tropospheric ozone is a novel selection driver, although yet insufficiently explored. Some studies demonstrate substantial intra-specific phenotypic and inter-specific variation in ozone resistance [17,18]. Therefore, changes in any stage of species life cycle, produced by increasing ambient ozone concentration, could modify species interactions and eventually impact on plant community structure and functioning [8]. In fact, it has already been documented that prolonged exposure to carbon dioxide increases species seed number and changes seed quality, thus altering community composition in the long term [45].

Ozone was found to produce changes in seed germination. The differences between ozone selection treatments could only be seen when germination levels were increased by storage at 75% RH/25 °C and 5%/5 °C. The observed increase in germination levels, particularly after lower storage temperatures, is consistent with evidence for other summer annuals. These species require an after-ripening period with low temperatures to reduce seed dormancy levels, allowing for germination waves during spring [32]. *S. arvensis* seed dormancy has long been documented [34,35], but the effect of tropospheric ozone over this seed trait has not been previously explored. Even though van Staden et al. [46] reported that scarification did not affect *S. arvensis* seeds germination, our results clearly show that this treatment increased germination dramatically. Actually, maximum seed germination was observed under alternating temperature treatments when previously stored at 75% RH/5 °C plus scarification. The physical dormancy level depended on the two-way interaction between storage condition and incubation temperatures. Instead, ozone selection treatments marginally influenced seeds dormancy level, thus determining the rate of dormancy release. Seeds from 90 ppb ozone selection treatment showed the highest level of germination under our testing conditions, which suggests that they had fewer germination requirements. Variation between ozone selection treatments in the germination response and dormancy level suggest that the four-year ozone exposure period acted as a natural selection factor over this weedy species. Also, the maternal environment could affect seed coat thickness, thus changing dormancy and germination [47].

Tropospheric ozone exerted a selection pressure on *S. arvensis* populations, resulting in three populations with different seed viability. Although seeds from the 90 and 120 ppb ozone selection treatments were less dormant, the viability level of these seeds in the soil surprisingly remained higher and for a longer period than seeds from 0 ppb ozone selection treatment, but depending on the experimental time. More interestingly, seeds from the three ozone selection treatments had higher initial viability and lower rate of mortality when they were produced by plants from the high concentration maternal ozone treatment. A differential synthesis of antioxidants may be the mechanism behind the prolonged seed viability in the soil. Seed cells have the capacity to synthesize antioxidant compounds, thus counteracting the oxidative effect of ROS (reactive oxygen species involved in seed ageing) and prolonging seed viability and persistence [48]. It is known that ozone increases ROS production in plants and consequently raises the antioxidants concentration in seeds and the whole plant [49]. Only those individuals that had the capability to produce enough antioxidant concentration may have survived and reproduced, producing seeds with thicker coats and higher antioxidant concentration. In this way, the population would adapt, not through a higher ozone tolerance, but through the selection of those individuals that produced seeds with the ability to remain alive in the soil for longer periods. However, when plants belonging to the 0 ppb ozone selection treatment were subjected to a short and high ozone exposure, they produced seeds with higher longevity than plants of the same ozone selection treatment grown at ambient ozone concentration. This shows that antioxidant production in seeds could also be induced by a short ozone exposure, that is, through an acclimation process (i.e. maternal effect). This has already been found in *Lolium* *multiflorum* seeds [50], supporting the idea that populations growing under high ozone concentrations, may produce seeds that stay alive in the soil for longer periods of time.

Global warming and increasing concentrations of tropospheric ozone are two major drivers of global change. Rising temperature has been documented to have a negative effect on seed dormancy, and thus, on soil seed bank persistence [25]. On the contrary, we found that the influence of previous ozone exposure may increase seed dormancy and persistence in the soil. The apparent opposite influence produced by rising temperatures and ozone on these seed traits, suggest that further efforts are needed to understand the interaction between these factors and their effects on species persistence in novel ecological scenarios. Indeed, a possible synergism between these two drivers might be taking place considering that tropospheric ozone does not only exert a direct oxidative effect on living organisms, but it also has the capability to act as a greenhouse gas contributing to rising temperatures. High ozone concentrations used for both the long and short-term exposure experiments are realistic values already measured in various regions of the world. This suggests that if the positive effect of ozone on seed persistence is repeated in several weedy species, rural areas where ozone concentrations are usually higher could be affected by an increase in the presence and persistence of weeds affecting crop production. This negative effect on crops could be increased by the direct oxidative effect of ozone on their photosynthetic organs, reducing the ability of crops to compete with weeds [12,30]. In the current global change scenario in which food production is threatened, further efforts will be needed to eradicate undesirable species from the fields.

## Supporting Information

Appendix S1 **Dynamic of soil temperature and seed water content during the Soil seed bank experiment.** Description of the method implemented to measure seed water content and soil temperature in the soil seed banks. A description of the data obtained is also provided.
(DOC)

Figure S1 **Dynamics of soil temperature (ºC) and seed water content (%) related to burial time (days).** Mean, minimum and maximum soil temperature indicated by solid, broken, and dotted lines respectively (left y-axis) and seed water content indicated by grey bars (right y-axis), taken at 5 cm depth during the whole experimental period.
(TIF)
