# Supplementary material for: Ozone Exposure of a Weed Community Produces Adaptive Changes in Seed Populations of Spergula arvensis
Source: PLoS One. 2013 Sep 26;8(9):e75820. doi: 10.1371/journal.pone.0075820 (PMC3784398; doi:10.1371/journal.pone.0075820)
Supplement: Appendix S1 — Dynamic of soil temperature and seed water content during the Soil seed bank experiment. Description of the method implemented to measure seed water content and soil temperature in the soil seed banks. A description of the data obtained is also provided. (DOC) [file pone.0075820.s001.doc]

**Appendix S1**

**Dynamic of soil temperature and seed water content during the Soil seed bank experiment**

To estimate seed moisture, five nylon mesh bags containing *S. arvensis* seeds were randomly placed in each seed bank and one mesh bag per soil seed bank was extracted every month along the experiment duration. Seeds were weighed (fresh weight) and later dried at 60 ºC for 48 hours, being re-weighed (dry weight) afterwards. Seed water content was expressed in percentage on fresh weight basis. During the first month of the experiment, soil seed banks maximum, minimum and mean temperatures were measured using a logger placed five centimeters deep. Likewise, during the whole experiment, the maximum, minimum and mean air temperatures were registered with a logger installed in the field. Given the direct relationship between soil and air temperature, after the first month, the former was estimated by using linear regressions relating maximum, minimum and mean air temperatures recorded along all the experimental period with those measured for the soil during the first month [1].

Seed water content (SWC) was highly variable along the experimental period (Fig. S1). The SWC was about 15%, ranging between ~5% (at the beginning) and ~25% depending on the experimental time (Fig. S1). The soil temperatures were high, but they experienced a slight fall in accordance with the proximity to autumn and winter months. Temperature showed important daily amplitude depending on season. In summer, maximum temperatures were between 35 and 41 ºC and minimum temperatures were between 24 and 29 ºC. In the autumn-winter period, maximum temperatures were between 34 and 38 ºC and minimum ones were between 19 and 26 ºC (Fig. S1).

**References**

1. Benech-Arnold RL, Ghersa CM, Schlichter TM (1987) Simulación de flujo de calor en el suelo. Evaluación de métodos numéricos. Ciencia del Suelo 5(2): 105-115.
